# Complete Sequence of a New Bipartite Begomovirus Infecting Cotton Plants in Brazil

**DOI:** 10.1128/genomeA.00661-13

**Published:** 2013-11-07

**Authors:** Mariana Martins Severo de Almeida, Sona Jain, Paulo Augusto Vianna Barroso, Lúcia Vieira Hoffmann, Monaliza Gomes de Lucena, Renato de Oliveira Resende, Alice Kazuko Inoue-Nagata

**Affiliations:** Universidade de Brasília, Brasília, Brazila; Universidade Federal de Sergipe, São Cristóvão, Brazilb; Embrapa Cotton, Santo Antônio de Goiás, Goiás, Brazilc; Universidade Estadual da Paraíba, Paraíba, Brazild; Embrapa Vegetables, Brasília, Brazile

## Abstract

Begomoviruses are plant viruses responsible for severe losses in important crops, such as beans, cassavas, tomatoes, and cotton, around the world. Here, we report the first full-genome sequence of a bipartite begomovirus species collected from cotton plants in Brazil.

## GENOME ANNOUNCEMENT

The begomoviruses (family *Geminiviridae*, genus *Begomovirus*) are circular single-stranded plant DNA viruses, encapsidated in icosahedral geminate particles, and are 18 by 30 nm. The viral genome is characterized by one (monopartite) or two (bipartite) (DNA-A and DNA-B) segments each around 2.6 kb in length. Although begomoviruses are particularly devastating to cotton plants in Asia (1–3), no such reports have been observed in Brazil.

Five cotton plants exhibiting chlorotic spots, interveinal chlorosis, and leaf distortion were collected at Paraiba state in 2009. The begomovirus infection was confirmed by PCR using the universal primers PAL1v1978 and PAR1c496 (4). The full length DNA-A and DNA-B genomes were cloned into pBlueScript vector (Stratagene, La Jolla, California) after rolling circle amplification (5) and digestion with the single cutting enzyme XbaI. Sequencing was performed at Macrogen, Inc. (South Korea) by primer walking. The contigs were assembled using the Staden 4 package (6), and the pairwise distances were calculated by MEGA 5 (7).

From a total of five samples, three similar clones corresponding to the DNA-A segment and six similar clones corresponding to the DNA-B were obtained, and the clones B012-6 and B012-2, corresponding to DNA-A and DNA-B, respectively, from sample B012 were chosen for further analysis. The cloned DNA-A and DNA-B genomes are 2,670 and 2,650 nucleotides long, respectively. They share a common region (CR) of 247 nucleotides with 98% identity, indicating that they are possibly cognate DNA segments of the same virus. The CR of both components contains the known nonanucleotide sequence TAATATTAC that is conserved in geminiviruses. They also contain the likely iteron sequence GGAGT and a G-rich region sequence, known as the “G box.” The genome organization is identical to that of other bipartite begomoviruses.

The DNA-A sequence of this virus, named cotton chlorotic spot virus (CCSV), shares 77.8% nucleotide identity with tomato common mosaic virus (ToCMV) (accession no. NC_018350), the most related begomovirus, and the DNA-B shares 67.8% nucleotide identity with tomato yellow vein streak virus (ToYVSV) (accession no. NC_010950).

### Nucleotide sequence accession numbers.

The nucleotide sequences have been deposited into GenBank under the accession no. KF358470 and KF358471.

## References

[1] Briddon RW 2003 Cotton leaf curl disease, a multicomponent begomovirus complex. Mol. Plant Pathol. 4:427–434 20569402 10.1046/j.1364-3703.2003.00188.x

[2] Briddon RW Markham PG 2000 Cotton leaf curl virus disease. Virus Res. 71:15–159 10.1016/s0168-1702(00)00195-7 11137169

[3] Mansoor S Bedford I Pinner MS Stanley J Markham PG 1993 A whitefly-transmitted geminivirus associated with cotton leaf curl disease in Pakistan. Pak. J. Bot. 25:105–107

[4] Rojas MR Gilbertson RL Russel DR Maxwell DP 1993 Use of degenerate primers in the polymerase chain reaction to detect whitefly-transmitted geminiviruses. Plant Dis. 77:340–347

[5] Inoue-Nagata AK Albuquerque LC Rocha WB Nagata T 2004 A simple method for cloning the complete begomovirus genome using the bacteriophage φ29 DNA polymerase. J. Virol. Methods 116:209–211 14738990 10.1016/j.jviromet.2003.11.015

[6] Staden R 1996 The staden sequence analyzes package. Mol. Biotechnol. 5:233–241 8837029 10.1007/BF02900361

[7] Tamura K Peterson D Peterson N Stencher G Nei M Kumar S 2011 MEGA5: molecular evolution genetics analysis using maximum likelihood, evolutionary distance, and maximum parsimony methods. Mol. Biol. Evol. 28:2731–2739 21546353 10.1093/molbev/msr121 PMC3203626

